# ALLFTD: Characterization of Frontotemporal Lobar Degeneration (FTLD) Disease Trajectories through Longitudinal Assessment

**DOI:** 10.1002/alz.093231

**Published:** 2025-01-03

**Authors:** Hilary W. Heuer, Leah K. Forsberg, Carly T. Mester, Tyler Kolander, Noah Johnson, Danielle Brushaber, Howard J. Rosen, Brad F. Boeve, Adam L. Boxer, ALLFTD Consortium

**Affiliations:** ^1^ Memory and Aging Center, UCSF Weill Institute for Neurosciences, San Francisco, CA USA; ^2^ Department of Neurology, Mayo Clinic, Rochester, MN USA; ^3^ Mayo Clinic, Rochester, MN USA

## Abstract

**Background:**

The ALLFTD (ARTFL‐LEFFTDS Longitudinal Frontotemporal Lobar Degeneration) study is an NIH‐funded effort to prepare for clinical trials in sporadic (s‐FTLD) and familial (f‐FTLD) FTLD syndromes by characterizing cohorts, developing new clinical trial outcome measures, and evaluating disease progression. To understand disease trajectories in the context of potential preventative or disease‐modifying therapeutic agents, comprehensive evaluation across multiple time‐points is crucial.

**Method:**

ALLFTD evaluates participants with FTLD spectrum disorders (bvFTD, svPPA, nfvPPA, FTD‐ALS, CBS, PSP), with strong family histories of FTLD, or known FTLD‐associated genetic variants within the family. ALLFTD has expanded to include 28 expert sites in North America conducting clinical and neuropsychological evaluations, MR Imaging, blood draws, and CSF collection in willing participants, providing detailed data on participant status and disease progression. Participants are asked to return for annual evaluations. The central data management staff assist sites in retention efforts by providing monthly reports of follow‐up visits that are pending, due, or overdue, based on a 15‐month period from previous visit.

**Result:**

Since enrollment began in January 2020, ALLFTD has conducted 1363 baseline visits, including 339 participants previously enrolled in ALLFTD’s predecessor studies (ARTFL and LEFFTDS). Excluding individuals not anticipated to return due to disease progression (123), participant death (55), or recent enrollment (<15 months; 318), ALLFTD has a retention rate of 83% for a second visit despite early enrollment occurring during COVID restrictions. Retention rates for third and fourth visits are even higher (90% and 97% respectively), suggesting a high degree of engagement in longitudinal participants. Including historic data from ARTFL and LEFFTDS, >1000 participants have multiple visits.

**Conclusion:**

Longitudinal assessment of clinical and biomarker profiles in f‐FTLD and s‐FTLD can inform models of disease onset and progression, allowing analysis of disease trajectories and identification of the most sensitive clinical outcome measures over time. ALLFTD has been successful at recruiting and retaining a large active cohort to obtain detailed clinical and biomarker characterizations; data are available by request.

